# Dysregulated IL-1β Secretion in Autoinflammatory Diseases: A Matter of Stress?

**DOI:** 10.3389/fimmu.2017.00345

**Published:** 2017-04-04

**Authors:** Sonia Carta, Claudia Semino, Roberto Sitia, Anna Rubartelli

**Affiliations:** ^1^Cell Biology Unit, IRCCS AOU San Martino-IST, Genova, Italy; ^2^Unit of Protein Transport and Secretion, Division of Genetics and Cell Biology, IRCCS San Raffaele Scientific Institute, Milan, Italy; ^3^Vita-Salute San Raffaele University, Milan, Italy

**Keywords:** autoinflammatory syndromes, endoplasmic reticulum stress, IL-1β, inflammation, monocytes, NLRP3 inflammasome, oxidative stress, toll-like receptor

## Abstract

Infectious and sterile inflammation is induced by activation of innate immune cells. Triggering of toll-like receptors by pathogen-associated molecular pattern or damage-associated molecular pattern (PAMP or DAMP) molecules generates reactive oxygen species that in turn induce production and activation of pro-inflammatory cytokines such as IL-1β. Recent evidence indicates that cell stress due to common events, like starvation, enhanced metabolic demand, cold or heat, not only potentiates inflammation but may also directly trigger it in the absence of PAMPs or DAMPs. Stress-mediated inflammation is also a common feature of many hereditary disorders, due to the proteotoxic effects of mutant proteins. We propose that harmful mutant proteins can induce dysregulated IL-1β production and inflammation through different pathways depending on the cell type involved. When expressed in professional inflammatory cells, stress induced by the mutant protein activates in a cell-autonomous way the onset of inflammation and mediates its aberrant development, resulting in the explosive responses that hallmark autoinflammatory diseases. When expressed in non-immune cells, the mutant protein may cause the release of transcellular stress signals that trigger and propagate inflammation.

## Introduction

The term “autoinflammation” (1) groups syndromes with different etiologies characterized by systemic inflammation in the absence of detectable infections and/or autoimmunity. Autoinflammatory diseases are disorders of the innate immune system, sharing recurrent episodes of fever, rash, joint pain, neutrophilia, and increased inflammatory markers. Most of them are monogenic, and the causative gene relates to the innate immune system. Examples are MEFV/pyrin in familial Mediterranean fever (FMF), TNFRSF1A/TNF receptor type 1 in TNF receptor-associated periodic syndrome (TRAPS), and nucleotide-binding domain, leucine-rich-containing family, pyrin domain-containing 3 (NLRP3) in cryopyrin-associated periodic syndromes (CAPS) (2).

The reversal of clinical symptoms in CAPS patients upon treatment with recombinant IL-1 receptor antagonist (*Anakinra*) or with IL-1β blocking agents (e.g., *Canakinumab*, a neutralizing antibody) provided compelling *ex adjuvantibus* evidence for the key role of IL-1β (3). The efficacy of anti-IL-1 drugs suggested that “gain-of-function” mutations in NLRP3, a central component of the inflammasome, cause uncontrolled IL-1β production, in turn responsible for the severe inflammatory symptoms (4, 5). Less expectedly, the same drugs displayed strong therapeutic effects also in autoinflammatory diseases, where the causative gene is not directly involved in IL-1β production and regulation (2, 3). A representative case is TRAPS, a disease characterized by recurrent episodes of long-lasting fever, pain, and fasciitis. Despite TRAPS is caused by mutations in p55 TNF receptor type I, patients showed no or modest response to TNFα inhibition (6), whereas IL-1β-blocking agents have high efficacy (7). These observations suggest that the presence of a mutated protein in inflammatory cells, independently from its function, activates mechanisms converging on dysregulated IL-1β secretion.

In this perspective article, we propose a pro-inflammatory role for cell stress and the responses it elicits in some hereditary diseases, and suggest that stress is a central player in the pathophysiology of autoinflammatory disorders, due to its presence in innate immune cells.

## Stress and Inflammation

Inflammation is traditionally defined as a reaction to infectious or sterile injuries, aimed at recruiting molecules and cells of the immune system to the tissue where the damage is taking place and restoring homeostasis. Inflammation is initiated by activation of pattern recognition receptors on inflammatory cells, by two subclasses of ligands responsible for infectious and sterile inflammation, respectively (8, 9): pathogen-associated molecular patterns and damage-associated molecular patterns (PAMPs and DAMPs). The former are part of pathogens, while the latter are components of cells or extracellular matrix released or degraded upon cell and tissue damage. Additional factors concur in determining the onset, duration, and intensity of inflammatory responses. Among these, particularly important is cell stress due to starvation, enhanced metabolic demand, cold or heat, altered proteostasis. The most common and well studied cell stresses are endoplasmic reticulum (ER) stress and oxidative stress that are counteracted by highly conserved responses. These responses share common traits, for example, eIF2α phosphorylation, with transient translational inhibition and transcriptional activation of chaperones and antioxidants (10). This integrated stress response prevents the toxicity caused by misfolded proteins [named “proteotoxicity” (10)] and limits reactive oxygen species (ROS)-based vicious circles. If excessive or prolonged, however, virtually all stress responses become maladaptive and induce inflammation due to activation of chemokine genes or, in case of cell damage, release of DAMPs that recruit inflammatory cells (11).

Oxidative stress is due to excessive production and/or deficient detoxification of ROS. These can be abundantly generated by mitochondria during oxidative phosphorylation (12) and by flavoenzymes like NADPH oxidases (NOX) (13). In cells of the innate immune system, phagocytosis and toll-like receptor (TLR) triggering activate NOX to produce abundant H_2_O_2_ (14). H_2_O_2_ is released into phagosomes to clear microorganisms and induces pro-inflammatory cytokines and inflammation: however, it may generate oxidative stress (13, 14). ROS are also produced in the ER as a by-product of oxidative protein folding, particularly in conditions of ER stress, which elicit the unfolded protein response (UPR) (15, 16). ER stress occurs when misfolded proteins accumulate in the secretory pathway, and also during infections, lipid unbalance, and other metabolic defects (15). UPR, a complex set of intracellular signaling pathways, has evolved to respond to protein misfolding and restore ER homeostasis. In addition, UPR signaling has a recognized role in immunity and inflammation (16). Oxidative and ER stresses are intimately linked: the former can induce misfolding of secretory proteins impacting disulfide bond formation. On the other hand, ER stress leads to ROS production (17). In concert with ROS, a prolonged UPR can induce NF-κB-mediated chemokine production and recruit inflammatory cells. In turn, PAMP or DAMP can potentiate the UPR (16).

These vicious circuits are evident in many chronic disorders such as type 2 diabetes (18), obesity (19), lung respiratory disease (20), inflammatory bowel disease (21), non-alcoholic fatty liver disease (22), and cancer (23).

Also in many hereditary diseases, the mutant protein may alter proteostasis: if this occurs, stress and inflammation are induced. For example, in cystic fibrosis, different mechanisms contribute to the inflammatory lung disease that is the major cause of morbidity and mortality in patients affected by this disease. Firstly, the mutated cystic fibrosis transmembrane conductance regulator (CFTR) protein cannot fold properly into the ER lumen, causing accumulation of misfolded CFTR aggregates, ER stress, and UPR. In turn, UPR activates NF-κB inducing production of chemokines, such as IL-8, that recruit polymorphonuclear leukocytes (PMN). PMN increase the oxidative burden in the lung, with generation of ROS that amplify the production of IL-8 thus locally increasing PMNs (24). Moreover, upregulation of ROS inhibits autophagy with consequent accumulation of protein aggregates and lung inflammation (25). Finally, the mutant CFTR transporter is unable to channel antioxidants into the airways: oxidative stress is worsened and concurs to the hyperinflammatory phenotype (24).

In Duchenne muscle dystrophy, due to the defect of dystrophin, oxidative stress and UPR-activated NF-κB interactively promote fiber necrosis. Recruited macrophages generate inflammatory cytokines and ROS, thereby triggering vicious inflammatory waves (26, 27).

**Figure 1:**
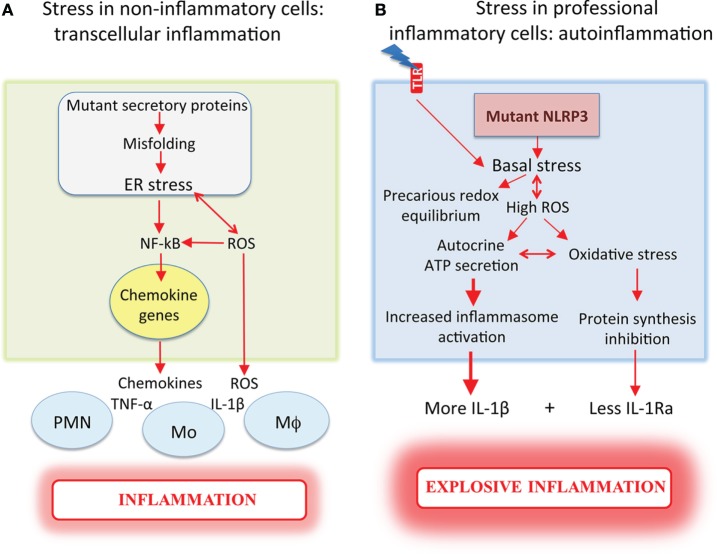
**Mutant proteins induce more severe inflammation when expressed in professional inflammatory cells**. **(A)** Non-inflammatory cells (e.g., epithelia or muscle), which express mutant proteins that undergo aberrant folding in the ER, exhibit ER stress and increased ROS, and promote NF-κB-mediated chemokine induction. The release of chemokines recruits inflammatory cells that secrete pro-inflammatory cytokines, ultimately causing inflammation. **(B)** Inflammatory cells from cryopyrin-associated periodic syndrome patients, which express mutated NLRP3, display cell stress with high reactive oxygen species (ROS) and antioxidant levels resulting in a precarious redox equilibrium that is deranged by toll-like receptor (TLR) stimulation. The high ROS levels facilitate autocrine ATP secretion, with increased and accelerated IL-1β secretion. When the antioxidant responses collapse, oxidative stress occurs with inhibition of protein synthesis responsible for the decrease of IL-1Ra secretion. Dysregulated cytokine production results in explosive inflammation. ROS are released in both conditions, triggering loops of amplification of stress and inflammation.

Differently from autoinflammatory disorders, in these cases, the mutant protein, being synthesized by epithelia or muscle, determines the release of stress signals that recruit leukocytes ultimately causing inflammation. These signals include small molecules like ROS and antioxidants, and proteins such as thioredoxin (28) and chemokines (as described above for cystic fibrosis, 24), which induce inflammation transcellularly, i.e., by recruiting and activating other cells (Figure 1A). When instead it is a professional inflammatory cell that produces a proteotoxic mutant, inflammation is generated in a cell-autonomous way and the onset, development, and outcome of it will be much worse for the host (Figure 1B).

## Stress in CAPS Monocytes

This hypothesis is supported by the observation that monocytes from CAPS patients, which express mutated NLRP3 molecules, display redox distress even before PAMP stimulation. Why mutant NLRP3 causes stress is unclear. A possible explanation is that it changes the affinity for the other components of the inflammasome complex (4), causing a disruption of the cytosolic homeostasis with induction of stress and integrated stress responses (10). Whatever the reason of NLRP3-induced stress, CAPS monocytes have higher basal ROS levels than monocytes from healthy donors but also higher expression of antioxidant systems (29–31) that allow them to maintain the redox homeostasis despite their stressed state. This equilibrium is, however, precarious, and CAPS monocytes can easily be induced to overreact, through pathways that largely depend on extracellular ATP, the most common inflammasome-activating signal (32). ATP is released by injured tissues, activated platelets, and other cells through pathways that are still ill defined (32). Unlike other pro-inflammatory cells, however, human monocytes do not need ATP from external sources. The accumulation of ROS upon TLR triggering (33) induces them to secrete ATP (34) that autocrinally or paracrinally stimulates cognate purinergic receptors (P2X7R) at the cell surface (32, 34). The ensuing lower intracellular [K^+^] induces inflammasome assembly and IL-1β secretion (35). The higher ROS levels in CAPS monocytes following TLR triggering facilitate ATP release that increases and accelerates IL-1β secretion (31) (Figure 1B).

Cell stress also decreases the threshold for IL-1β processing and secretion: minute amounts of TLR agonists, that in healthy monocytes are sufficient to trigger pro-IL-1β synthesis but not its processing and secretion, drive large amounts of IL-1β release in CAPS monocytes (31). Probably owing to their “pre-activated state,” small doses of TLR agonists increase ROS, inducing abundant ATP release, and IL-1β processing and secretion (31). This circuit explains why small traumas or infections that go undetected in healthy subjects can cause severe inflammatory manifestations in CAPS patients.

**Figure 2:**
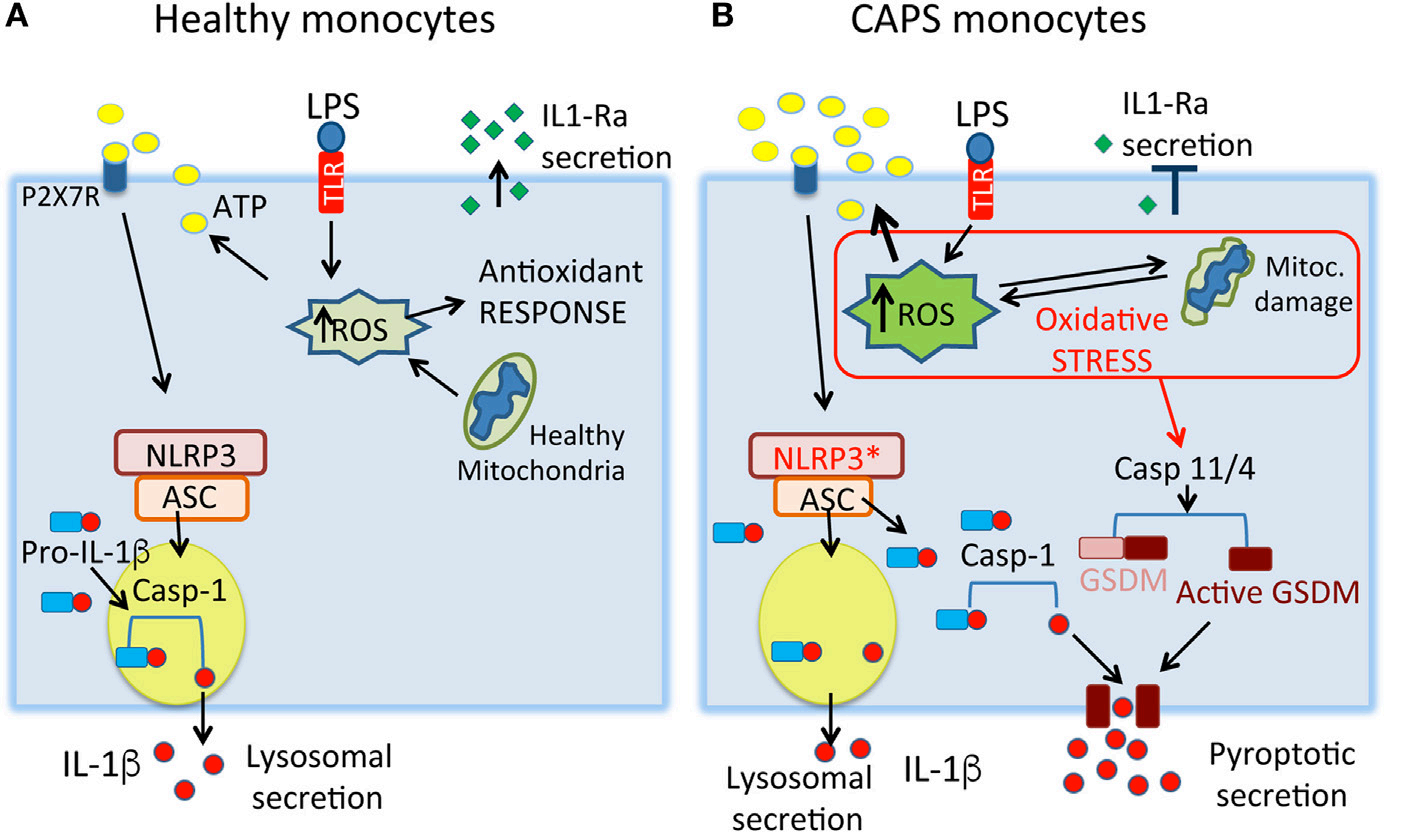
**A model for stress-mediated cytokine secretion in cryopyrin-associated periodic syndromes (CAPS) monocytes**. **(A)** In healthy monocytes, toll-like receptor (TLR) stimulation induces the production of low amounts of reactive oxygen species (ROS), rapidly neutralized by the antioxidant response. The ROS-induced ATP release is low, resulting in processing and secretion of little amounts of IL-1β through secretory lysosomes. The anti-inflammatory cytokine IL-1Ra is produced, contributing to switch off the inflammatory response. **(B)** In CAPS monocytes, small doses of TLR agonists induce a strong increase of ROS resulting in release of large amounts of endogenous ATP and IL-1β. The state of stress may trigger pyroptotic secretion of IL-1β through activation of caspase-11/4 that cleaves gasdermin D (GSDMD) generating a toxin-like N-terminal peptide that forms pores on the plasma membrane. Mature IL-1β, cleaved by the NLRP3 inflammasome, will be released through gasdermin D-formed pores. Later, failure of antioxidant response and mitochondria dysfunctions lead to severe oxidative stress, with impaired production of IL-1Ra. NLRP3*, mutated NLRP3.

The effects described above occur soon after TLR stimulation. In later phases, the precarious redox equilibrium of CAPS monocytes is broken as antioxidant responses collapse. CAPS monocytes display damaged mitochondria (30), a further indication of the presence of oxidative stress [(12), Figure 2]. Interestingly, mitochondria are normal in CAPS lymphocytes, which do not express NLRP3, and in monocytes from healthy donors, which express wild-type NLRP3 (30), suggesting that mutant NLRP3 is indeed the causative agent of the oxidative stress. In this crucial phase, stress impacts also the production of IL-1Ra, normally secreted by activated monocytes a few hours after IL-1β to limit inflammation [(30), Figure 1]. Thus, deficient IL-1Ra production likely concurs in increasing the severity of the disease. Highlighting the dangerous stress-inflammation liaisons, insufficient IL-1Ra production may depend on eIF2α phosphorylation. Indeed, TLR-activated monocytes from CAPS patients, but not from healthy donors, display attenuated protein translation (30). Thus, IL-1ra mRNA is transcribed but stress prevents translation. Once more, oxidative and ER stress appear to be linked because IL-1Ra secretion is restored by antioxidants.

## Hyper-Stimulated Healthy Monocytes Recapitulate the Behavior of CAPS Monocytes

The above observations suggest that the increased IL-1β/IL-1Ra ratio in CAPS depends on the synergistic effects of NLRP3 mutations and stress. Combinations of PAMPs that stimulate surface and intracellular TLRs (LPS, R848, zymosan) were then used to induce a CAPS-like stress state in healthy monocytes (36). When given alone, each TLR agonist triggered the secretion of IL-1β and IL-1Ra by healthy monocytes. When provided simultaneously, however, they induced a superstimulation resulting in enhanced secretion of IL-1β but impaired release of IL-1Ra (36). The underlying molecular mechanisms are similar to those described in CAPS monocytes (29–31): super-stimulation induces ROS accumulation, responsible of the massive ATP release and IL-1β secretion, and of the consequent oxidative stress leading to inhibition of IL-1Ra production, despite normal IL-1Ra mRNA levels. Antioxidants restore IL-1Ra release by super-stimulated healthy monocytes, confirming the role of oxidative stress and recapitulating the phenotype of CAPS monocytes (36). However, the latter are constitutively stressed by the mutation (37) so that stimulation with low doses of a single TLR agonist strongly increases stress that drives prompt and abundant IL-1β secretion and, in a second phase, lowers IL-1Ra (30, 31). In healthy monocytes with balanced basal redox state (29, 36), instead, multiple TLR co-stimulation is needed to cause cell stress and derange the normal cytokine network (36). These observations may suggest that, in CAPS, mutations in NLRP3 are more important indirectly, triggering and enduring stress, than directly activating inflammasome.

## Different Mechanisms for IL-1β Secretion: Does Stress Determine the Pathways of Secretion?

Since IL-1β is a potent and potentially dangerous mediator of inflammation, its production is tightly controlled virtually at all levels, including post-translationally (38, 39). IL-1β is synthesized as an inactive precursor, pro-IL-1β, and processed mainly by caspase-1, which in turn must be activated by the inflammasome. Only mature, 17 kDa IL-1β is then secreted. The underlying mechanisms are still poorly understood. Indeed, IL-1β secretion has been a problem for cell biologists, since it was shown that the cytokine lacks a secretory signal sequence (40). Initially, a popular view was that the cytokine was released by dying cells. However, further studies demonstrated that secretion of mature IL-1β is an active process, requires living cells, avoids the ER-Golgi route, and involves secretory lysosomes (41–44). In addition to this pathway, recent studies revealed another route for IL-1β release, involving pyroptosis. This is a highly inflammatory form of programmed cell death, which has been proposed to mediate IL-1β secretion under condition of strong stimulation such as infection with intracellular pathogens (45–48). According to this model of secretion, stressful stimuli (e.g., intracellular LPS) activate caspase-11 (the mouse homologous of human caspase-4). In turn, caspase-11 cleaves gasdermin D, generating toxin-like peptides that form pores on the plasma membrane, which allow secretion of mature IL-1β, but not of the 33 kDa precursor (48, 49). It remains to be determined how the pores guarantee transport selectivity.

The two pathways are not mutually exclusive, and the choice of lysosomal or pyroptotic secretion may depend on the strength of pro-inflammatory signals (Figure 2). Mild stimuli, such as low amounts of PAMPs triggering surface bound TLRs, would induce the less efficient but more regulated lysosomal pathway. Accordingly, low doses of LPS induce pro-IL-1β synthesis, but not ATP secretion (31): in the absence of a second trigger, therefore, pro-IL-1β is degraded by lysosomal proteases (31, 42) preventing unnecessary inflammation. Stronger stimuli, such as intracellular infections with gram-negative bacteria (45) could instead induce pyroptosis, causing massive release of IL-1β and possibly DAMPs, and dysregulated cytokine production (36).

Support to this hypothesis comes from our preliminary observations that human monocytes display more IL-1β-containing lysosomes when stimulated with LPS alone than with three agonists simultaneously triggering extra- and intracellular TLRs (unpublished results). Moreover, only in monocytes stimulated with extracellular LPS alone, do drugs interfering with lysosomal function modulate IL-1β secretion. Conversely, caspase-4 inhibitors block IL-1β release only in super-stimulated monocytes (unpublished results).

It is possible that the secretory lysosome-mediated mechanism is more active in low pathogen load or small trauma, as a way to restore the homeostasis. Differently, the pyroptosis-mediated secretion would intervene in severe inflammatory responses, characterized by strong or multiple stimuli such as it may occur in sepsis (50), diabetes (51) or cancer (52).

The ongoing stress could also determine the route of IL-1β secretion. Owing to the high ROS levels that favor ATP release, we predict that CAPS monocytes utilize preferentially the pyroptotic pathway. Accordingly, caspase-4 inhibition blocks IL-1β secretion by CAPS monocytes stimulated with a single TLR agonist, a condition that neither involves caspase-4 nor induces pyroptosis in healthy monocytes (unpublished) (Figure 2).

## Cell-Autonomous Proteotoxic Stress in Monocytes Increases IL-1β Secretion in Autoinflammatory Diseases

Increased IL-1β secretion has been reported *in vitro* by monocytes from other autoinflammatory diseases, including FMF (53), TRAPS (54) hyperimmunoglobulinemia D syndrome (55), pyogenic sterile arthritis, pyoderma gangrenosum and acne (PAPA) (56), and also in the milder NLRP-12-associated periodic syndrome (57). As introduced above, anti-IL-1β therapies are the standard of care in these syndromes (58), suggesting that IL-1β is a key culprit. Nonetheless, the links between the mutated gene and IL-1β secretion are elusive. Remarkably, in these diseases, the mutant genes are expressed by monocytes that are under stress (53, 57, 59–61). It is tempting to speculate that stress and the ensuing responses converge to induce excessive IL-1β secretion, possibly switching from lysosomal to pyroptotic secretion (Figure 2). The consequences on disease severity are many, since pyroptosis-mediated secretion would alter the networks of pro- and anti-inflammatory cytokine production.

Stress-induced hyperinflammatory response may occur in other inherited diseases that are not (yet) classified as autoinflammatory diseases. This is the case of chronic granulomatous disease (CGD), a disorder linked to mutations in NOX2. Because of these mutations, phagocytes of CGD patients fail to produce ROS with consequent deficiency in bactericidal activity and increased susceptibility to infections (62). In addition, and consistent with the evidence that CGD is associated with increased inflammasome activation (63–65), patients often develop hyperinflammatory traits. Moreover, *Anakinra* induced significant clinical improvement in two cases with colitis (66). Thus, CGD was defined as a potentially lethal combination of immunodeficiency and excess inflammation (67), most likely due to cell-autonomous stress responses. Likewise, evidence is accumulating for a role of stress and inflammation in the pathogenesis of Gaucher disease, the inherited deficiency of lysosomal glucocerebrosidase (68). Monocyte/macrophages from these patients display increased secretion of IL-1β that depends on increased inflammasome activation, in turn due to the impaired autophagy secondary to the lysosomal enzyme deficiency (68). A further example is mucopolysaccharidosis type I, where, in innate immune cells, stress induced by lysosomal storage defects can upregulate immunity-related genes. In turn, these may be responsible for the severe inflammation-dependent pathologies observed in patients (69).

## Conclusion and Perspectives

In essence, we propose that stress hallmarks monocytes from patients affected by autoinflammatory syndromes (and possibly other inherited diseases) that express mutant proteins not necessarily directly involved in IL-1β production. Stress induces inflammation and is, therefore, a key pathogenetic factor in these diseases. The stress levels contribute to determine the severity of the disease, and so do individual differences in resistance to oxidative stress, as proposed for chronic inflammation-mediated diseases such as diabetes (70). Accordingly, we showed clear correlations among basal stress, ongoing antioxidant responses, and disease severity in two CAPS patients sharing the same NLRP3 mutation (31). Extending these concepts, we suggest that a similar stress-related mechanism may be operative in other genetic diseases, where the mutant protein is present in monocytes and inflammation participates to disease progression. Considering that individual tolerance plays a major role (71), improving the responses to stress represents a promising therapeutic opportunity for these serious diseases.

## Author Contributions

SC, CS, RS, and AR designed, wrote, and approved the final manuscript.

## Conflict of Interest Statement

The authors declare that the research was conducted in the absence of any commercial or financial relationships that could be construed as a potential conflict of interest.

## Abbreviations

CAPS, cryopyrin-associated periodic syndromes; DAMPs, damage-associated molecular patterns; ER, endoplasmic reticulum; IL, interleukin; NLRP3, nucleotide-binding domain, leucine-rich-containing family, pyrin domain-containing 3; NOX, NADPH oxidases; PAMPs, pathogen-associated molecular patterns; ROS, reactive oxygen species; TLRs, toll-like receptors; TRAPS, TNFRSF1A/TNF receptor type 1 in TNF receptor-associated periodic syndrome; UPR, unfolded protein response.
